# Chirality-Induced Spin Selectivity in Composite Materials: A Device Perspective

**DOI:** 10.1021/acs.accounts.4c00077

**Published:** 2024-04-30

**Authors:** Seyedamin Firouzeh, Md Anik Hossain, Juan Manuel Cuerva, Luis Álvarez de Cienfuegos, Sandipan Pramanik

**Affiliations:** † Department of Electrical and Computer Engineering, University of Alberta, Edmonton, Alberta T6G 1H9, Canada; ‡ Universidad de Granada, Departamento de Química Orgánica, Unidad de Excelencia Química Aplicada a Biomedicina y Medioambiente, C. U. Fuentenueva, Avda. Severo Ochoa s/n, E-18071 Granada, Spain; § Instituto de Investigación Biosanitaria ibs., Avda. De Madrid, 15, E-18016 Granada, Spain

## Abstract

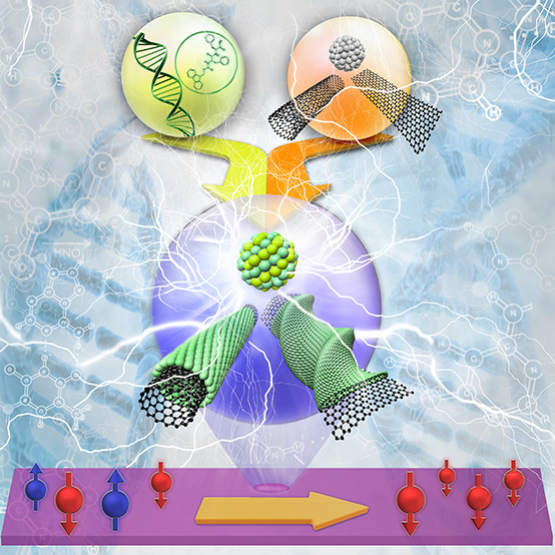

Magnetism is an area of immense fundamental and technological importance. At the atomic level, magnetism originates from electron “spin”. The field of nanospintronics (or nanoscale spin-based electronics) aims to control spins in nanoscale systems, which has resulted in astronomical improvement in data storage and magnetic field sensing technologies over the past few decades, recognized by the 2007 Nobel Prize in Physics. Spins in nanoscale solid-state devices can also act as quantum bits or qubits for emerging quantum technologies, such as quantum computing and quantum sensing.

Due to the fundamental connection between magnetism and spins, ferromagnets play a key role in many solid-state spintronic devices. This is because at the Fermi level, electron density of states is spin-polarized, which permits ferromagnets to act as electrical injectors and detectors of spins. Ferromagnets, however, have limitations in terms of low spin polarization at the Fermi level, stray magnetic fields, crosstalk, and thermal instability at the nanoscale. Therefore, new physics and new materials are needed to propel spintronic and quantum device technologies to the true atomic limit. Emerging new phenomena such as chirality induced spin selectivity or CISS, in which an intriguing correlation between carrier spin and medium chirality is observed, could therefore be instrumental in nanospintronics. This effect could allow molecular-scale, chirality controlled spin injection and detection without the need for any ferromagnet, thus opening a fundamentally new direction for device spintronics.

While CISS finds a myriad of applications in diverse areas such as chiral separation, recognition, detection, and asymmetric catalysis, in this focused Account, we exclusively review spintronic *device* results of this effect due to its immense potential for future spintronics. The first generation of CISS-based spintronic devices have primarily used chiral bioorganic molecules; however, many practical limitations of these materials have also been identified. Therefore, our discussion revolves around the family of chiral *composite* materials, which may emerge as an ideal platform for CISS due to their ability to assimilate various desirable material properties on a single platform. This class of materials has been extensively studied by the organic chemistry community in the past decades, and we discuss the various chirality transfer mechanisms that have been identified, which play a central role in CISS. Next, we discuss CISS device studies performed on some of these chiral composite materials. Emphasis is given to the family of chiral organic-carbon allotrope composites, which have been extensively studied by the authors of this Account over the past several years. Interestingly, due to the presence of multiple materials, CISS signals from hybrid chiral systems sometimes differ from those observed in purely chiral systems. Given the sheer diversity of chiral composite materials, CISS device studies so far have been limited to only a few varieties, and this Account is expected to draw increased attention to the family of chiral composites and motivate further studies of their CISS applications.

## Key References

- RahmanMd. W.; Mañas-TorresM. C.; FirouzehS.; CuervaJ. M.; Álvarez de CienfuegosL.; PramanikS.Molecular Functionalization and Emergence of Long-Range Spin-Dependent Phenomena in Two-Dimensional Carbon Nanotube Networks. ACS Nano2021, 15 ( (12), ), 20056–20066.34870421 10.1021/acsnano.1c07739(1) Demonstration of the CISS effect in two-dimensional carbon nanotube (CNT) networks, functionalized by chiral peptides. The CISS signal is tunable by the chemical composition of the peptides and survives micron-scale distances at low temperatures.
- RahmanMd. W.; Mañas-TorresM. C.; FirouzehS.; Illescas-LopezS.; CuervaJ. M.; Lopez-LopezM. T.; Álvarez de CienfuegosL.; PramanikS.Chirality-Induced Spin Selectivity in Heterochiral Short-Peptide-Carbon-Nanotube Hybrid Networks: Role of Supramolecular Chirality. ACS Nano2022, 16 ( (10), ), 16941–16953.36219724 10.1021/acsnano.2c07040(2) The effect of supramolecular chirality on CISS. Supramolecular chirality and, hence, the CISS signal can be controlled by adding small amounts of a secondary chiral material, which acts as a dopant and offers an additional mechanism to control the CISS signal.
- HossainM. A.; Illescas-LopezS.; NairR.; CuervaJ. M.; Álvarez de CienfuegosL.; PramanikS.Transverse Magnetoconductance in Two-Terminal Chiral Spin-Selective Devices. Nanoscale Horizons2023, 8 ( (3), ), 320–330.36740957 10.1039/d2nh00502f(3) The measurement of CISS in a transverse geometry, indicating the presence of a spin component transverse to the current, which is also independent of electromagnetochiral anisotropy. Onsager’s reciprocity is shown to be valid in this geometry.
- FirouzehS.; Illescas-LopezS.; HossainM. A.; CuervaJ. M.; Álvarez de CienfuegosL.; PramanikS.Chirality-Induced Spin Selectivity in Supramolecular Chirally Functionalized Graphene. ACS Nano2023, 17 ( (20), ), 20424–20433.37668559 10.1021/acsnano.3c06903PMC10604086(4) Demonstration of the CISS effect in chiral peptide-functionalized graphene layers.

## 1 Introduction and Overview

**Figure 1:**
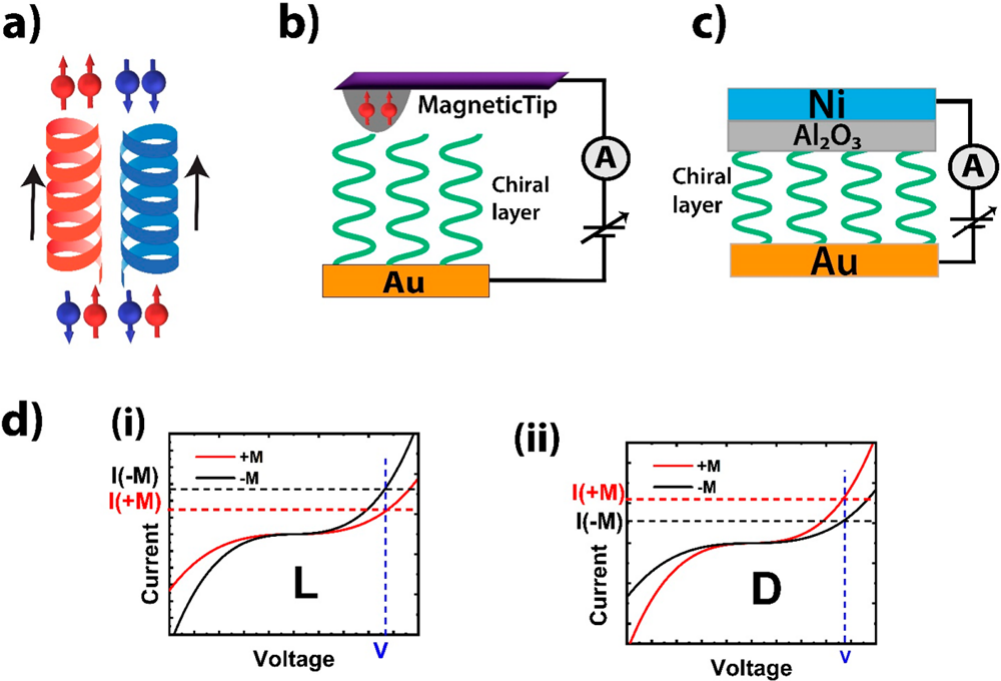
(a) Schematic description of the CISS effect. Transmission of spin-unpolarized electrons through a chiral medium results in a chirality dependent spin polarization, with spins polarized parallel or antiparallel to the velocity (indicated by arrow). (b) CISS measurement geometry using Au film and a magnetic AFM tip, probing few chiral molecules. (c) CISS measurement geometry using a vertical sandwich structure, probing a large ensemble of chiral molecules. (d) Chirality dependent current asymmetry (*I*_±*M*_) observed in two-terminal CISS experiments.

Chirality induced spin selectivity (CISS) refers to the phenomenon in which spin-unpolarized carriers, after transmission through a chiral medium, acquire a chirality dependent spin polarization (Figure 1a).^5,6^ This effect has profound implications in condensed matter, device physics, and spintronics, as well as in chiral electrocatalysis and enantiomer separation.^5,6^ Specific to solid-state spintronic devices, CISS may allow the electrical generation and detection of spin-polarized carriers at a molecular level, thus opening a novel direction for future nanospintronics.

Extensive reviews on experimental and theoretical aspects of CISS are available in refs (5) and (6). Here we will summarize only the most common CISS phenomenologies to provide sufficient context for the subsequent discussions. Briefly, CISS has been observed in photoemission experiments, in which spin-unpolarized electrons emitted from a Au substrate acquire significant spin polarization after passing through a chiral ds-DNA layer.^7^ CISS has also been reported in device setups, in which a ds-DNA molecule was contacted by a Au particle and a ferromagnetic spin detector (Ni).^5^ Spin-unpolarized electrons passing through Au become spin-polarized after transmission through the ds-DNA molecule. Depending on the Ni magnetization orientation (±*M*), two different current values (*I*_±*M*_) are obtained (Figure 1d), indicating a net spin polarization of the incoming electrons. CISS *magnetoresistance* (MR) can be defined as (*I*_+*M*_ – *I*_–*M*_)/(*I*_+*M*_ + *I*_–*M*_), which is often described as “spin polarization” or “spin selectivity” in the literature.^5^ This quantity changes sign for the opposite chirality.^5^

Two device geometries are commonly studied:^5^ (a) setups using a magnetic atomic force microscopy (AFM) tip (mCP-AFM) (Figure 1b), probing very few chiral molecules in a nanometer-scale domain, and (b) setups with a vertical sandwich structure (Figure 1c), probing a large ensemble of molecules. In the former case, the device current is highly sensitive to the tip–molecule contact, and to address the spatial and temporal variations, such studies typically measure hundreds of current–voltage scans in each magnetic configuration and compare the average responses. These studies generally report a high degree of spin selectivity, although such configurations are not suitable as practical devices. In the second case, spin selectivity is generally much weaker, presumably due to sample inhomogeneity over a larger area, the presence of spin-independent leakage paths via pinhole shorts, and poor control of the interface quality.

The simplicity of the two-terminal MR devices described above belies the underlying conceptual complexity. For example, (a) the two-terminal MR is expected to be zero in the linear bias range according to Onsager’s reciprocity, leading to significant controversy regarding the measured CISS signals.^8^ (b) The role, if any, of electromagnetochiral anisotropy (EMChA) on CISS, especially in a device setup with magnetic contacts, is highly debated.^9^ (c) Spin polarization or spin selectivity, as defined above, represents CISS MR, and extraction of carrier spin polarization is not straightforward.^10^ (d) While the CISS MR generally appears in a longitudinal geometry with current parallel to the magnetic field, in some systems, a transverse CISS MR was observed.^3^ (e) Temperature dependence of MR responses varies significantly depending on the measurement setup (Figure 1b,c). For example, the AFM geometry (Figure 1b) typically shows CISS response at room temperature,^11,12^ whereas the vertical sandwich geometry (Figure 1c) often shows low-temperature response^13,14^ and, in some cases, room-temperature response.^11,12^

Despite the above open questions, the two-terminal vertical CISS MR devices discussed above remain quite relevant due to their structural simplicity and resemblance with more established spintronic devices such as spin valves and giant- or tunnel-magnetoresistance devices.^15^ However, due to the poor conductivity of common chiral organics (DNA, amino acids, etc.), they are not ideal materials for spintronic devices. Also, limited work has been done on planar multiterminal CISS devices, presumably due to the high resistance of these chiral molecules, which makes this geometry impractical. Nevertheless, such studies are essential for not only obtaining a deeper understanding of the CISS effect but also opening up new device research opportunities.^8^

It is therefore clear that alternative chiral materials with improved material properties and electronic conductivity need to be investigated for CISS device applications, and composite chiral materials, which combine the high electron mobility of inorganic materials with the chirality of organics, can play a crucial role in this regard. In section 2, we discuss various chiral composites and chirality transfer mechanisms that are essential for the CISS effect. CISS device studies on these materials are discussed in sections 3 and 4. We conclude in section 5 with future perspectives of the CISS effect from the viewpoint of composite chiral materials and device applications. In this Account, our discussion mainly revolves around two-terminal spintronic devices. For other applications of CISS, the reader is referred to broader reviews such as ref (5).

## 2 Chirality in Composite Materials

Most of the chiral composite materials developed for CISS use enantiopure organic compounds combined with generally achiral, inorganic, metallic, or carbon allotropes. Chirality transfer occurs from the organics to the inorganics, and this mechanism is critical, as it determines the CISS effect. Four principal chirality transfer mechanisms are discussed below.

### 2.1 Enantioselective Surface Distortion by Chiral Molecules

**Figure 2:**
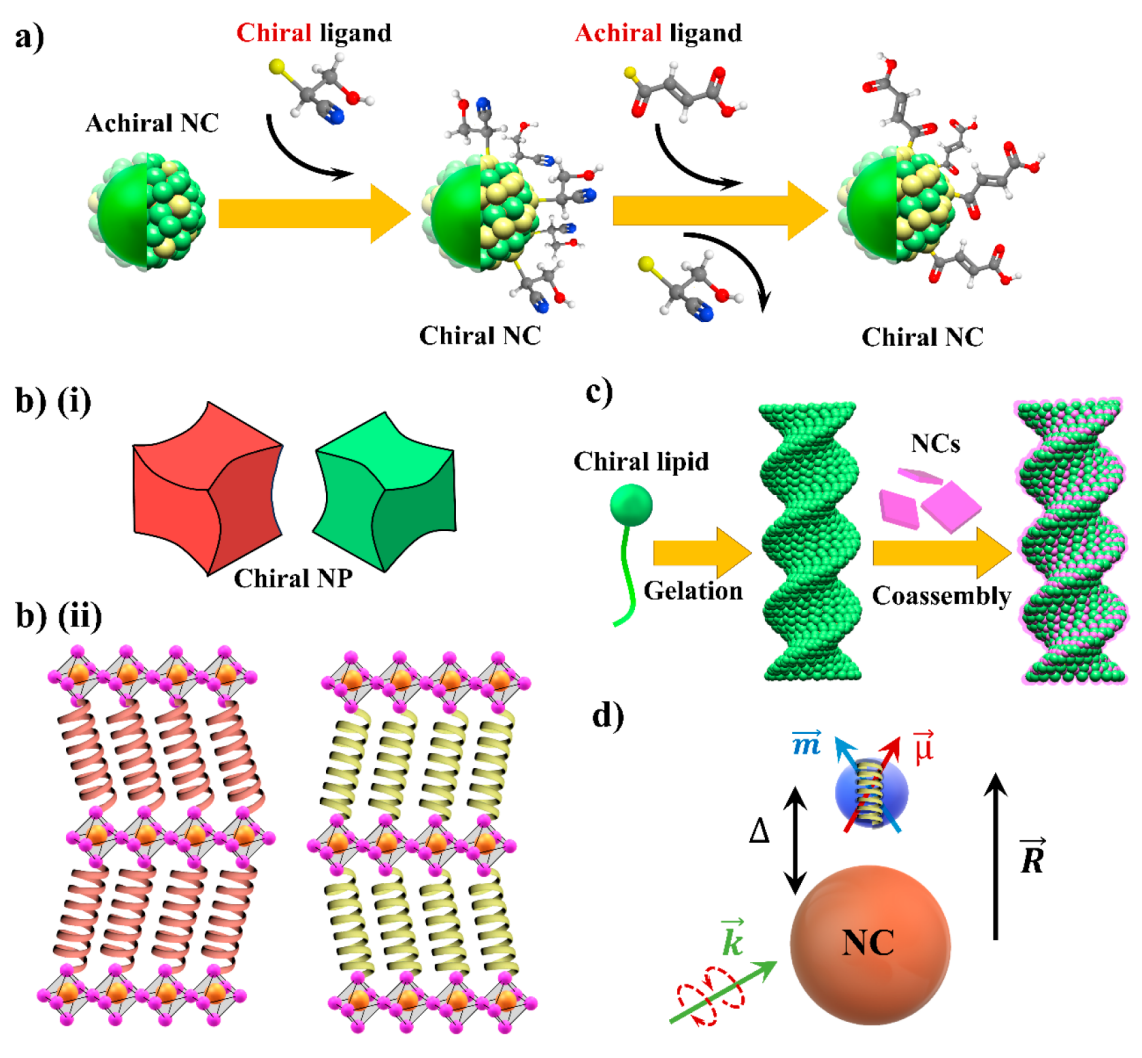
Various chiral composite materials and chirality transfer mechanisms. (a) Enantioselective surface distortion by chiral molecules. (Adapted from ref (18). Copyright 2009, American Chemical Society.) The surface of the achiral nanocrystal (NC) can be made chiral by attachment of chiral ligands on the surface. The chirality of the NC surface is indicated by the arrangement of the yellow spheres. In some cases, subsequent replacement of the chiral ligand by an achiral ligand preserves the surface chirality of the nanocrystal, as shown in the schematic. This is the so-called “chiral memory effect”. (b) (i) Schematic of chiral crystals (adapted from ref (24) under a CC license). The original straight edges of the nanoparticles are twisted in opposite directions, depending on the chirality of the molecule. (b) (ii) Schematic of 2D hybrid chiral perovskites with chiral molecules inserted in the inorganic framework. Conceptually similar structures are obtained by intercalating chiral molecules in the interlayer regions of 2D van der Waals materials. (c) Schematic of inducing chirality in perovskite nanocrystals via co-assembly in chiral gels (adapted from ref (30) with permission from Wiley VCH). Chiral lipid could form a gel via chiral assembly. If perovskite NCs are added during this process, they can co-assemble and follow the chirality of the gel structure, which induces chirality in the NCs. (d) Schematic description of chiral molecule and achiral nanocrystal coupled via Coulombic interactions. (Adapted from ref (32). Copyright 2010, American Chemical Society).

In this strategy, the surface of inorganic nanocrystals (e.g., metallic nanoparticles (NPs) such as Au, inorganic nanocrystals (NCs) such as CdX (X = S, Se, and Te), and perovskites) is covered by enantiopure organic ligands. This modifies the surface via a chemical bond between the chiral ligands and the metallic centers on the surface of the NCs, which causes a transfer of chirality to the crystal surface and related electronic states. This strategy was first reported using Au NPs and sulfur containing l-glutathione.^16^ These chiral surface-functionalized Au NPs showed strong chiroptical responses, proving that the electronic structure of the metal could be easily tuned by the capping molecules. Later, a similar strategy was explored with Cd-based inorganic nanocrystals, prepared by microwave irradiation in the presence of organic chiral ligands such as l/d-penicillamine and l/d-cysteine methyl ester.^17,18^ Results showed that NC cores were achiral and the surface was chiral due to the attached chiral organic ligand. In contrast with the chiral Au NPs,^16^ the surface of the inorganic particle became permanently chiral, thus rendering these NCs intrinsically chiral even after removing the chiral ligand. This is known as the *chiral memory effect* (Figure 2a).

Chiral perovskite NCs have also been obtained following a similar protocol.^19^ In this case, CsPb(I/Br)_3_ NCs capped with oleylamine were microwave irradiated in the presence of different amounts of *R*/*S*-1,2-diaminocyclohexane. As above, the chiroptical properties of the resulting perovskites were retained even after removing the chiral ligand; therefore, the organic ligand not only induced a transfer of chirality by surface adsorption but also exerted a chiral transformation in the inorganic crystal.

### 2.2 Formation of Chiral Crystals by Chiral Molecules

Some inorganic crystals, such as quartz, Se, Te, β-AgSe, α-HgS, etc., have intrinsic chiral structures.^20,21^ NCs of these materials are expected to show stronger chiroptical properties than those obtained by surface modification since, in this case, the whole particle and not only the surface are arranged in a chiral structure. The main drawback is that in the absence of any chiral stimulus, racemic mixtures are obtained. Ref (22) showed that enantiopure α-HgS can be obtained by a colloidal precipitation method in the presence of an enantiopure additive such as penicillamine. Sulfur containing penicillamine binds to Hg^2+^ during crystal nucleation and growth, inducing the preferred formation of one enantiomorph. The same strategy has also allowed enantiomorphic Te and Se crystals.^23^ This approach achieves a higher hierarchical level of chirality with respect to the previous approach.

Ref (24) reported a similar strategy, in which incubation of achiral Au NP seeds with l/d amino acids and peptides gave rise to intrinsically chiral particles (Figure 2b, panel i), with their handedness being governed by the chirality of the organic additive. The induction of chirality in bulk Au and Ag via a doping effect was reported in ref (25).

Chiral organic–inorganic crystals were obtained by the combination of lead halides and a chiral primary amine (*R*/*S*-methylbenzylamine). These structures arranged in one-dimensional (1D) chains and two-dimensional (2D) layers, in which the organic molecules are inserted between the inorganic halides (Figure 2b, panel ii).^26^ These 2D layered perovskites exhibit circular dichroism (CD), in which the sign is determined by the chirality of the amine.^26^ Recently, chiral molecules have been intercalated in 2D van der Waals materials such as MoS_2_, TiS_2_, TaS_2_, etc., which imparts chirality to the composite system.^27,28^

### 2.3 Supramolecular Chirality by Self-Assembly

The self-assembly of small molecules into higher-order aggregates gives rise to a new type of chirality known as supramolecular chirality. Chirality is a property of the global system and not of the individual component, being sufficient in the absence of a symmetry plane or an inversion center. Therefore, chiral supramolecular aggregates can be developed by using achiral monomers. In this case, a symmetry breaking event must take place. Solid surfaces can be viewed as such kinds of symmetry breaking inductors once 2D chirality is considered. However, racemic mixtures are usually obtained. To avoid this drawback, the achiral entities can be placed in a chiral environment dictated by organic-based supramolecular arrangements. Two different strategies have been mainly studied: in the first strategy, the nanoparticles are formed in an existing chiral network, which acts as a supramolecular template; the other strategy employs a process of co-assembly, in which the particle is incorporated in the chiral network at the same time this is formed by a process of self-assembly. In both cases, the nanoparticle suffers a chirality transfer process, being surrounded and covered by a chiral supramolecular network. This strategy has been used to grow Au nanoparticles in helical supramolecular structures and to obtain gold nanorods embedded in chiral fibers.^29^ By a similar process of co-assembly, perovskite NCs have been embedded in a chiral network formed by self-assembly (Figure 2c).^30^ As described in section 4, we have used the strategy of co-assembly to induce chirality in carbon nanotubes (CNTs) and graphene by promoting the self-assembly of enantiopure aromatic short peptides in the presence of these carbon-based materials.^1−4,31^

### 2.4 Electronic Interactions between Chiral and Achiral Materials

Beyond surface modifications, chiral molecules can interact through space with NCs via complex dipole and multipole Coulombic interactions (Figure 2d) without requiring surface or crystal distortions and with the consequent appearance of new CD bands. It has been shown that the NCs induce a change in the electromagnetic field of the chiral molecule, and the chiral molecule also induces a chiral current inside the crystal.^32^ The latter mechanism explains the CD signal at the plasmon frequency. It is worth noting that this type of interaction can be exerted simultaneously with the previously mentioned ones.^33^

## 3 Spin Selectivity in Composite Organic–Inorganic/Metallic Materials

Some of the composite chiral materials discussed above exhibit the CISS effect in electronic transport experiments. Ref (12) studied chiral cysteine capped CdSe quantum dots (∼2 nm) using both the mCP-AFM geometry as well as the vertical thin-film sandwich structure, discussed earlier in section 1. The former shows a stronger spin selective response than the latter, as discussed previously. The MR response is found to be opposite compared to self-assembled monolayers of cysteine, which correlates with the sign of the CD signal, indicating that electron conduction is intimately related to the chirality of the system. For larger (∼6 nm) quantum dots, the CISS effect is negligible, which again correlates with the decreasing CD response with size. This is presumably because the chirality transfer effect is weakened with an increasing quantum dot size. Finally, the CISS MR was found to be almost independent of temperature. Although the issue of Onsager’s reciprocity was not directly addressed in this work, the data seem to indicate the validity of this principle in the linear range.

Organic–inorganic chiral perovskites, discussed above, are promising candidates for spintronics because of (a) long spin relaxation times, (b) strong spin–orbit coupling (SOC), (c) tunable Rashba splitting, and (d) chemically tunable optical and electrical properties.^34^ Lu et al. showed the CISS effect in solution-processed polycrystalline 2D chiral organic–inorganic lead iodide perovskite films using mCP-AFM.^14^ Spin selectivity, as defined in section 1, was found to be ∼86%. In contrast, in the sandwich geometry, this material showed <1% MR, only at low temperatures (∼10 K). While the smaller MR in the sandwich geometry is consistent with the chiral CdSe study discussed above, the temperature dependence of the MR signal is different in these two chiral systems.

A review of the CISS effect in chiral hybrid perovskites is available in ref (34). In summary, thin films of these materials typically show a large (∼90%) spin selectivity in vertical mCP-AFM measurements. As shown schematically in Figure 2b, panel ii, these materials have a 2D layered structure, in which chiral organic molecules occupy the space between two neighboring inorganic layers, and in a typical vertical transport setup, the charge carriers travel sequentially from one type of layer to the other. Chirality dependent spin polarization is accrued during their transport through the chiral layers. The inorganic sublattice confers structural ordering in these samples, which, coupled with multiple chiral tunneling steps, is responsible for the large CISS response in all of these cases.

Besides chiral perovskites, other organic–inorganic or organic–metallic composites have been developed for spintronics. Huizi-Rayo et al. reported the synthesis of a chiral three-dimensional (3D) metal–organic framework (MOF) based on the lanthanide Dy(III) and the l-tartrate chiral ligand.^35^ The spin polarization of this material proved to be ideal, showing spin selectivity up to 100% at a bias voltage of 2 V measured by mCP-AFM. The CISS effect has been measured in self-assembled monolayers (SAMs) of helical lanthanide-binding peptides via spin-dependent electrochemistry. In this case, two different mechanisms for spin filtering, paramagnetism and chirality, are combined in a single molecule. Results showed a spin polarization of SP = −70 ± 10% in optimal conditions.^36^ Spin selectivity in self-assembled chiral coordinated monolayers of cysteine-Cu^2+^-alanine^37^ and in chiral metal–organic Cu(II) phenylalanine crystals^38^ have also been reported.

2D atomic crystals of transition metal dichalcogenides allow for the introduction of chiral molecules between the atomic layers without disturbing the crystallinity. Qian et al. studied the CISS properties of 2D atomic crystals of TaS_2_ and TiS_2_ intercalated with *R*/*S*-methylbenzylamine.^27^ Results showed a clear chirality dependent out-of-plane current with a spin polarization of more than 60% at 10 K. Bian et al.^28^ studied out-of-plane transport in multilayer MoS_2_ intercalated with chiral methylbenzylamine molecules. mCP-AFM measurements showed ∼75% spin polarization. Similar to the hybrid perovskites discussed above, due to out-of-plane electronic transport, the observed CISS effect arises purely due to the interlayer chiral molecules present in the transport path. While the 2D layers offer structural stability and robustness to the devices, which improve the CISS response, their planar electronic and spintronic properties remain largely untapped.

Metal oxides such as NiO_*x*_ and NiFeO_*x*_ coated with chiral molecules have been used in the electrocatalytic oxygen evolution reaction (OER).^39^ The chiral molecules have been found to enhance the OER activity via the CISS effect.^39^

Overall, in hybrid systems such as chiral hybrid perovskites and 2D intercalation compounds, the inorganic sublattice confers structural ordering to the devices, which improves the CISS effect, as discussed above. Such gainful integrations make hybrid systems promising for future CISS studies. Another strategy that combines carbon allotropes with chiral molecules is discussed below.

## 4 Spin Selectivity in Chiral Organic-Carbon Allotrope Composite Materials

**Figure 3:**
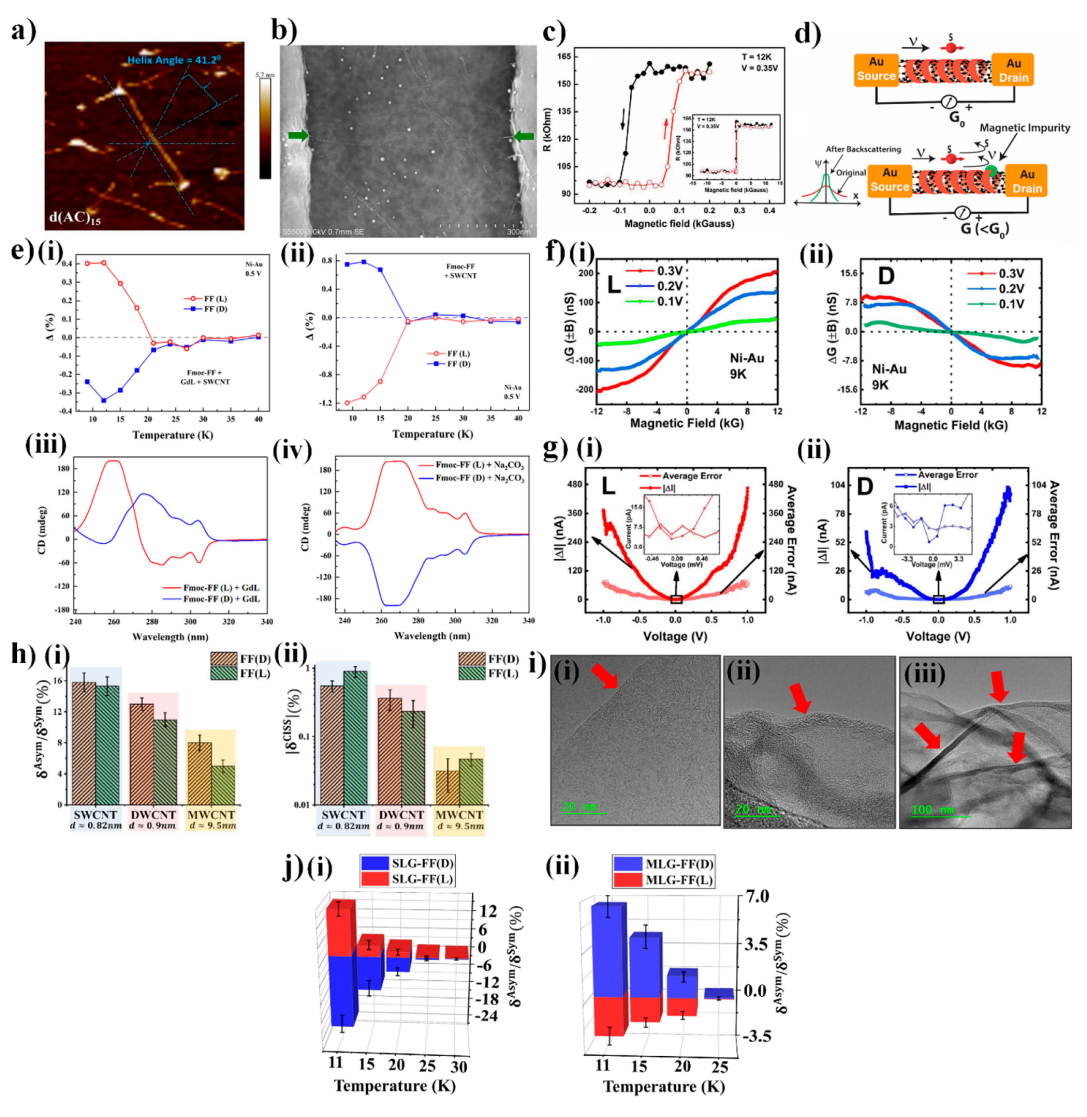
CISS effect in chiral organic–CNT/graphene composites. (a) Helical wrapping of CNTs by ssDNA (d(AC)_15_) strands. Periodic height modulation along the CNT axis indicates helical wrapping, and an estimation of the helix angle is shown. (Adapted from ref (47). Copyright 2020, American Chemical Society.) (b) Device geometry, in which a DNA-wrapped CNT is contacted by Ni–Au electrodes. The green arrows indicate the contact points. (c) MR behavior of DNA-wrapped CNTs. Two distinct conductance states are observed for two opposite orientations of Ni (spin detector) magnetization, implying spin filtering. (Figure 3b,c is reprinted from ref (44) with permission from Wiley VCH.) (d) Schematic description of the role of CISS on carrier localization. Backscattering (and hence carrier localization) is possible only when there is a magnetic impurity. (e) Role of chiral GDL on CISS. For Fmoc-FF, the addition of GDL changes the supramolecular chirality, with a concomitant change in the sign of the CISS signal. (Adapted from ref (2). Copyright 2022, American Chemical Society.) (f) CISS signal in a noncollinear geometry, with Ni spin detector magnetized transverse to the current direction. (g) The difference in current (Δ*I*) for two opposite Ni magnetizations approaches zero as bias approaches zero, validating Onsager’s reciprocity. (Figure 3f,g is reprinted from ref (3) with permission from the Royal Society of Chemistry.) (h) CISS signal as a function of CNT diameter (and hence spin–orbit interaction). (Reprinted from ref (31) with the permission of AIP Publishing.) (i) Unfunctionalized graphene layer with straight edge (image (i)) and chiral-functionalized graphene with folded edges (images (ii) and (iii)). (Adapted from ref (4). Copyright 2023, American Chemical Society.) (j) CISS effect in single-layer graphene (SLG) and multilayer graphene (MLG). (Adapted from ref (4). Copyright 2023, American Chemical Society.)

CNTs and graphene bind noncovalently with various organic molecules via π stacking and electrostatic interactions.^31,40^ The synthesis of CNT–DNA hybrids, in which DNA strands helically wrap CNTs (Figure 3a), has been reported in ref (41). Theoretical calculations have shown that such composites can act as spin filters.^42,43^ Electron transport occurs via the CNT channel, and the role of the DNA strands is to induce an inversion-asymmetric helicoidal electric field on the charge carriers. This electric field produces a strong Rashba spin–orbit interaction in the channel and polarizes the electron spins. In 2015, Alam et al. was the first to experimentally study spin filtering of ssDNA-wrapped single-wall (SW) CNT using d(GT)_15_ strands.^44^ The authors developed a planar device in which ssDNA-wrapped tubes were contacted between Au and Ni (spin detector) electrodes (Figure 3b). MR measurements showed that this system could act as a spin filter with spin polarization up to ∼74% at low temperatures (Figure 3c). Later, the authors showed that higher spin polarizations (∼80%) could be achieved by using ssDNA strands made only of thymine (TT)_15_^45^ or by using longer d(GT)_200_ strands.^46^ These results offered a new way to engineer spin polarization by changing the chemical composition of the DNA, opening the door to exploring other chiral compounds with varying chemical compositions. This was confirmed by studying the spin polarization of SWCNTs functionalized with d(AC)_15_ and d(CC)_15_ sequences and comparing the results with those measured before (d(GT)_15_ and d(TT)_15_).^47^ The binding energy is higher for T/G than for A/C,^47^ which resulted in lower CISS values for the d(AC)_15_- and d(CC)_15_-wrapped SWCNTs than the previously reported d(GT)_15_- and d(TT)_15_-wrapped SWCNTs.

The effect of CISS on carrier localization was analyzed in ref (48). CISS-induced spins tend to increase the carrier localization length. This is because the CISS spin polarization is coupled with the carrier’s momentum (either parallel or antiparallel, Figure 1a), and hence, in one-dimensional systems such as nanotubes, momentum flip must be accompanied by a simultaneous spin flip. Thus, backscattering is prohibited in the absence of spin-flip scattering events, which tends to increase the localization length (Figure 3d). This effect is manifested in the carrier transport properties in CNT–DNA systems.^47,48^

Rahman et al. further extended this concept by developing chiral 2D SWCNT networks functionalized with aromatic short peptides known to interact with CNTs.^1,40^ As discussed in section 2.3, SWCNTs were embedded in supramolecular peptide fibers obtained by self-assembly. Like ssDNA, peptide functionalization of CNTs is also noncovalent, and therefore, the degree of interaction is susceptible to the chemical composition of the peptides. SWCNTs functionalized with l/d-Fmoc-FF (fluorenylmethoxycarbonyl-diphenylalanine), l/d-Fmoc-AA (Fmoc-dialanine), and achiral Fmoc-GG (Fmoc-diglycine) showed different CISS behavior.^1^ SWCNTs functionalized with achiral Fmoc-GG did not give any detectable CISS signal, while SWCNTs functionalized with Fmoc-FF, which interacts strongly with CNTs due to the presence of a major number of aromatic groups, showed a CISS effect. Quite remarkably, the CISS signal survived length scales longer than 1 μm, and the CISS values obtained from these random CNT networks are similar to those of ordered chiral molecules under similar conditions.^5^

Next, the authors studied the influence of different chiral sources in the presence of CNTs.^2^ It is known that these types of aromatic short peptides can self-assemble by the application of different stimuli; the addition of the chiral molecule glucono-δ-lactone (GDL) is one such stimulus.^49^ The influence of GDL was studied with l/d-Fmoc-FF- and Fmoc-GG-functionalized SWCNTs (Figure 3e).^2^ Results showed that GDL interfered with only the process of self-assembly of short peptides, promoting the formation of chiral β-sheet secondary structures even in achiral Fmoc-GG peptides. The CISS effect originated from the supramolecular assembly of peptide fibers. Notably, the CISS effect was observed in achiral molecules (Fmoc-GG) through the formation of chiral supramolecular fibers mediated by GDL.

To investigate the existence of noncollinear CISS spin components and Onsager’s reciprocity, discussed in section 1, Hossain et al. studied CISS magnetoconductance (MC) in a planar two-terminal device with Ni/Au contacts in the presence of a transverse (out-of-plane) magnetic field *B*.^3^ The chiral layer contained SWCNTs functionalized with l/d-Fmoc-FF. Results showed that the CISS effect exists in transverse MC measurements (Figure 3f) and disappears in the linear response regime, proving the validity of Onsager’s relation (Figure 3g),^3^ at least in the context of CNT–chiral molecule hybrids. In this transverse geometry, the current *I* and magnetic field *B* are perpendicular, and hence, the CISS signal is devoid of any spurious MC effect due to electric magnetochiral anisotropy (EMChA).^9^ In contrast, in most of the CISS experiments discussed before, *B* is collinear with *I*, which may result in a nonzero EMChA contribution, and hence, MC values can be the result of both effects. The existence of a CISS signal in a transverse geometry where the EMChA effect is absent suggests that the CISS cannot be explained by invoking EMChA alone.

The role of spin–orbit coupling has also been explored using CNTs functionalized with chiral peptides.^31^ It is generally assumed that spin–orbit coupling of the chiral media must exist to observe the CISS effect.^6^ To test this hypothesis, in a previous work, we studied the role of spin–orbit coupling of the chiral media by changing the diameter of the CNTs, as it is well-known that the strength of spin–orbit coupling is inversely proportional to the CNT diameter.^31^ As discussed previously, transport occurs via CNTs, and it is the CNT spin–orbit interaction that the carriers experience. The attached molecules have negligible spin–orbit interaction due to their low atomic numbers, and transport does not occur through them; hence, they are not expected to contribute any significant spin–orbit interaction. The inversion asymmetric electrostatic helical potential due to the molecules, however, can enhance the native spin–orbit coupling via the Rashba effect, which is presumably responsible for the correlation between the CNT–DNA binding energy and CISS signal strength discussed earlier. Results showed that for a given chiral functionalization, the magnitude of the CISS signal correlates with the spin–orbit coupling strength of the nanotubes (Figure 3h). Interestingly, the nanotube diameter influenced the supramolecular chirality, which in turn determined the sign of the CISS signal.^31^

Graphene and graphene derivatives, such as graphene oxide (GO) and reduced GO (rGO), are promising for future electronics and spintronics.^50^ Nevertheless, investigation of the CISS effect in these materials is quite limited. To study this, Firouzeh et al. prepared chiral graphene sheets of different thicknesses, noncovalently functionalized with chiral l/d-Fmoc-FF.^4^ It was observed that graphene layers were distorted after chiral functionalization, acquiring a “conformational chirality” (Figure 3i). This effect, combined with other factors, was assumed to be responsible for the CISS signal. Again, the CISS signal was determined by the supramolecular chirality of the medium, which in this case was influenced by the graphene thickness (Figure 3j).

Comparing the CISS results observed in pure chiral molecules^5^ to those of the nanotube (or graphene) chiral hybrid materials discussed above, one finds some similarities as well as some differences. The differences are not unexpected since in the hybrid case, multiple materials are involved, and all of them are expected to contribute to the CISS signal. In terms of similarities, both systems show a chirality dependent asymmetric MR that correlates with the CD response. In addition, a change in the supramolecular chirality results in a concomitant change in the CISS signal, which is again consistent with the pure molecular case.^5^ In terms of magnitude or temperature dependence of device setups, we have not observed any systematic difference. The main difference is in the orientation of Ni magnetization. It appears that the strongest CISS signal occurs when the magnetization is transverse to the current, whereas in the purely molecular case, it is parallel. As discussed above, in these hybrid systems, the CISS signal strength correlates with the spin–orbit interaction; however, such correlation generally is not observed in pure chiral systems.^5,6^ In fact, the CISS effect observed in pure chiral systems is much larger than the weak spin–orbit interaction expected in organics, and hence, the presence of additional mechanisms is expected.^5,6^ Similarly, in these hybrid systems, Onsager’s reciprocity is shown to be valid, while this may not generally be the case for pure organics.^13^

## 5 Conclusions and Outlook

In conclusion, we have reviewed the chirality transfer mechanisms and CISS device results observed in various chiral composites. Given the sheer variety of chiral composites, CISS investigations on these systems are still at a nascent stage. For most of the CISS studies discussed in section 3, the spin-dependent effect arises from the chiral molecules rather than from the inorganic component, which keeps the true potential of these inorganic materials underutilized. Chiral composites based on carbon allotropes, as discussed in section 4, are remarkably different because charge transport primarily happens via the high mobility channels of CNT and graphene, and the properties of the channel (such as spin–orbit coupling) play a central role.

Due to the widespread applications of CNTs and graphene in emerging nanoelectronics, such an induced CISS effect could enable a variety of nanospintronic devices. In particular, these materials are amenable to lateral multiterminal geometries, which may allow CISS-based spin transistors. Multiterminal geometries will also permit devices based on the inverse CISS effect, i.e., injecting spins in a chiral material and generating chirality dependent electrical signals. Overall, in the long term, such studies are expected to expand spintronic device applications of CISS and may contribute to the development of quantum information processing using spin qubits without using any bulky micromagnet. In the short term, studies in this direction should address improving the interface quality, minimizing channel heterogeneity, and investigating multiterminal geometries to shed light on the physical mechanism of CISS in such systems.
